# Genetic Modification of *Mucor circinelloides* for Canthaxanthin Production by Heterologous Expression of β-carotene Ketolase Gene

**DOI:** 10.3389/fnut.2021.756218

**Published:** 2021-10-13

**Authors:** Tahira Naz, Junhuan Yang, Shaista Nosheen, Caili Sun, Yusuf Nazir, Hassan Mohamed, Abu Bakr Ahmad Fazili, Samee Ullah, Shaoqi Li, Wu Yang, Victoriano Garre, Yuanda Song

**Affiliations:** ^1^Colin Ratledge Center for Microbial Lipids, School of Agricultural Engineering and Food Science, Shandong University of Technology, Zibo, China; ^2^Department of Food Sciences, Faculty of Science and Technology, Universiti Kebangsaan Malaysia, Bangi, Malaysia; ^3^Department of Botany and Microbiology, Faculty of Science, Al-Azhar University, Assiut, Egypt; ^4^University Institute of Diet and Nutritional Sciences, Faculty of Allied Health Sciences, The University of Lahore, Lahore, Pakistan; ^5^Departamento de Genética y Microbiología (Unidad asociada al Instituto de Química Física Rocasolano-Consejo Superior de Investigaciones Científicas (IQFR-CSIC)), Facultad de Biología, Universidad de Murcia, Murcia, Spain

**Keywords:** *Mucor circinelloides*, *crgA*, canthaxanthin, β-carotene ketolase, overexpression

## Abstract

Canthaxanthin is a reddish-orange xanthophyll with strong antioxidant activity and higher bioavailability than carotenes, primarily used in food, cosmetics, aquaculture, and pharmaceutical industries. The spiking market for natural canthaxanthin promoted researchers toward genetic engineering of heterologous hosts for canthaxanthin production. *Mucor circinelloides* is a dimorphic fungus that produces β-carotene as the major carotenoid and is considered as a model organism for carotenogenic studies. In this study, canthaxanthin-producing *M. circinelloides* strain was developed by integrating the codon-optimized β-carotene ketolase gene (*bkt*) of the *Haematococcus pluvialis* into the genome of the fungus under the control of strong promoter *zrt1*. First, a basic plasmid was constructed to disrupt *crgA* gene, a negative regulator of carotene biosynthesis resulted in substantial β-carotene production, which served as the building block for canthaxanthin by further enzymatic reaction of the ketolase enzyme. The genetically engineered strain produced a significant amount (576 ± 28 μg/g) of canthaxanthin, which is the highest amount reported in *Mucor* to date. Moreover, the cell dry weight of the recombinant strain was also determined, producing up to more than 9.0 g/L, after 96 h. The mRNA expression level of *bkt* in the overexpressing strain was analyzed by RT-qPCR, which increased by 5.3-, 4.1-, and 3-folds at 24, 48, and 72 h, respectively, compared with the control strain. The canthaxanthin-producing *M. circinelloides* strain obtained in this study provided a basis for further improving the biotechnological production of canthaxanthin and suggested a useful approach for the construction of more valuable carotenoids, such as astaxanthin.

## Introduction

Canthaxanthin is a valuable ketocarotenoid, which is naturally present in algae, bacteria, and some fungi (1). Canthaxanthin is also commonly present in wild bird tissue, egg yolk, crustaceans, and fish at low levels. It was first isolated as a major coloring pigment from the edible mushroom *Cantharellus cinnabarinus*, from where it was named canthaxanthin (2). It is formed as an intermediate compound in β-carotene metabolisms to astaxanthin. It has more effective antioxidant potential than β-carotene (3) due to which it is regarded as one among the important xanthophylls of commercial significance and has extensive utilization in industries (4). In the poultry industry, it is used as a feed additive to obtain red color in egg yolks and skins (1), while in the cosmetics industry, it is used as a pigmenting agent for human skin applications.

In the past few decades, the demand for canthaxanthin has increased owing to its beneficial properties on human health such as anticancer, anti-inflammatory, anti-dermatosis, and coloring agent (5). It has been documented as an effective protective agent against skin cancers in the laboratory for the treatment of polymorphous light eruptions and idiopathic photodermatosis (6). It can effectively stimulate the immune defensive system as compared to other carotenoid species (7) and owns excellent medicinal properties for treating rashes and itching. It is anticipated that the natural canthaxanthin market will grow from 75 million to 85 million from 2018 to 2024 with a compound annual growth rate (CAGR) of 3.5% due to change in consumer preferences toward fermentation products instead of synthetic compounds (https://www.gminsights.com/industry-analysis/canthaxanthin-market).

Canthaxanthin is produced by plants, bacteria, microalgae, and halophilic archaeon (*Haloferax alexandrines)* in relatively low concentrations, hence these organisms are unable to compete economically with synthetic canthaxanthin (8). Synthetic canthaxanthin is produced from petrochemicals and is widely preferred due to its low cost. The applicability of plant-based canthaxanthin is also hampered due to geographical, and seasonal variation and marketing. However, microbial production of canthaxanthin by biotechnological approaches is a major development in comparison to chemical synthesis due to safety concerns connected with chemical-based canthaxanthin.

Microbial production of carotenoid using non-native platforms are appealing due to many drawbacks associated with natural hosts such as low abundance and more sophisticated growth requirements, etc. *Mucor circinelloides* is a well-known model organism to study the molecular background of carotene biosynthesis in zygomycetes due to the availability of the whole-genome sequence. *M. circinelloides* has been reported as a natural competent organism compared to other related species and also acts as a good host for heterologous gene expression (9, 10). These attributes make *Mucor* an amenable candidate for genetic modification for desired and high-value products. Although β-carotene is the principal carotenoid of *M. circinelloides*, it has also shown weak β-carotene hydroxylase activity, because it has shown the accumulation of zeaxanthin and β-cryptoxanthin in minute amounts (11). It is possible to produce new carotenoids such as xanthophylls in *Mucor* by exogenous expression of genes involved in carotenogenesis. For example, Papp et al. introduced canthaxanthin-producing genes *crtW* from *Paracoccus* sp. N81106 into *M. circinelloides* (12). The subsequent transformants produced canthaxanthin and astaxanthin but in low amounts, which was attributed to the low copy number of autonomously replicating plasmids. To solve this problem they introduced *crtW* gene into the genome of *Mucor* to get stable expression of the ketolase gene and obtained a higher amount of canthaxanthin with canthaxanthin as the principal carotenoid (13).

The present study attempted to construct a canthaxanthin-producing cell factory by integrating the β-carotene ketolase–encoded gene (*bkt*) from *Haematococcus pluvialis* inside the genome of *M. circinelloides* CBS 277.49. Since precursor accumulation is considered as a prerequisite for xanthophyll production so in the present study higher β-carotene availability as a substrate for β-carotene ketose enzyme was ensured by disrupting the *crgA* locus, which is a negative regulator of the carotene biosynthesis pathway. Then codon-optimized gene of *bkt* from *H. pluvialis* was overexpressed under the control of strong promoter *zrt1*. Integration can be forced by the transformation with linear fragments that have extensive homologous regions at their ends to direct homologous recombination and gene replacement (13). Thus, this study attempted to construct a mutant of *M. circinelloides* CBS 277.49 strain harboring the algal *bkt* gene for canthaxanthin production.

## Materials and Methods

### Strains, Media, and Growth Conditions

The double auxotroph (leucine and uracil) strain MU402 (14), derived from the wild-type *M. circinelloides* CBS277.49 was used as a recipient strain in all the transformation procedures. MU402 was grown on solid YPG media (3 g/L yeast extract, 10 g/L peptone, and 20 g/L glucose) at 28°C for 5–7 days to obtain spores for transformation. Transformants were grown on MMC media (10 g/L casamino acids, 0.5 g/L yeast nitrogen base (w/o amino acids), and 20 g/L glucose). Media were supplemented with uridine (200 μg m/L) when required. The pH was adjusted to 3 and 4.5 for colonial and mycelial growth, respectively.

For carotenoid extraction, all strains were initially grown by inoculating 100 μl of spore suspension (~10^7^ spores/mL) in 500 ml baffled flasks holding 150 ml Kendrick and Ratledge (K&R) medium for 24 h with 150 rpm at 28°C (15). Then these seed cultures were inoculated at 10% v/v into 2 L fermenters (BioFlo/CelliGen 115, New Brunswick Scientific, Edison, NJ, United States) containing 1.5 L modified K&R medium supplemented with 0.6 g/L of leucine to compensate leucine auxotrophy of transformants. Fermenters were maintained at 28°C with stirring at 700 rpm and aeration of 1 v/v per min and the pH was maintained at 6.0 by automatic addition of 2 M NaOH. All the transformants were cultivated for 4 days under continuous light. *Escherichia coli* strain DH5α was used for maintenance and amplification of recombinant plasmids and grown in lysogeny broth (LB) containing 100 μg/ml ampicillin at 37°C and 220 rpm (16).

### Construction of Recombinant Plasmids

According to the genomic data of *H. pluvialis* available at NCBI, the complete coding sequence of the β-carotene ketolase (GenBank accession No: AF534876.1) was synthesized by GeneWiz **(**GeneWiz, Suzhou, China). Codon optimization of the *bkt* genes was done according to the genome of *M. circinelloides* CBS 277.49 with the given GenBank accession number MZ020513 to increase the chances of successful expression. The codon-optimized sequence of *bkt* is given in Supplementary File 1. The gene sequence was provided in the modified vector, named pMAT1552+*bkt*. To disrupt *crgA* locus, a new basic plasmid was constructed and named pCRC53, which contained *pyrG* gene of *M. circinelloides* as a selection marker surrounded by 1 kb up- and down-stream of *crgA* sequences. The *pyrG* gene encodes orotidine 5′-phosphate decarboxylase and produces uridine to compensate uridine auxotrophy in *Mucor* and allows targeted genomic integration of the whole construct by homologous recombination. pCRC53 was used as a basic plasmid used for the generation of *bkt* overexpressing vector pCRC55. Plasmids used in the present study are listed in Supplementary Table S1.

**Figure 1:**
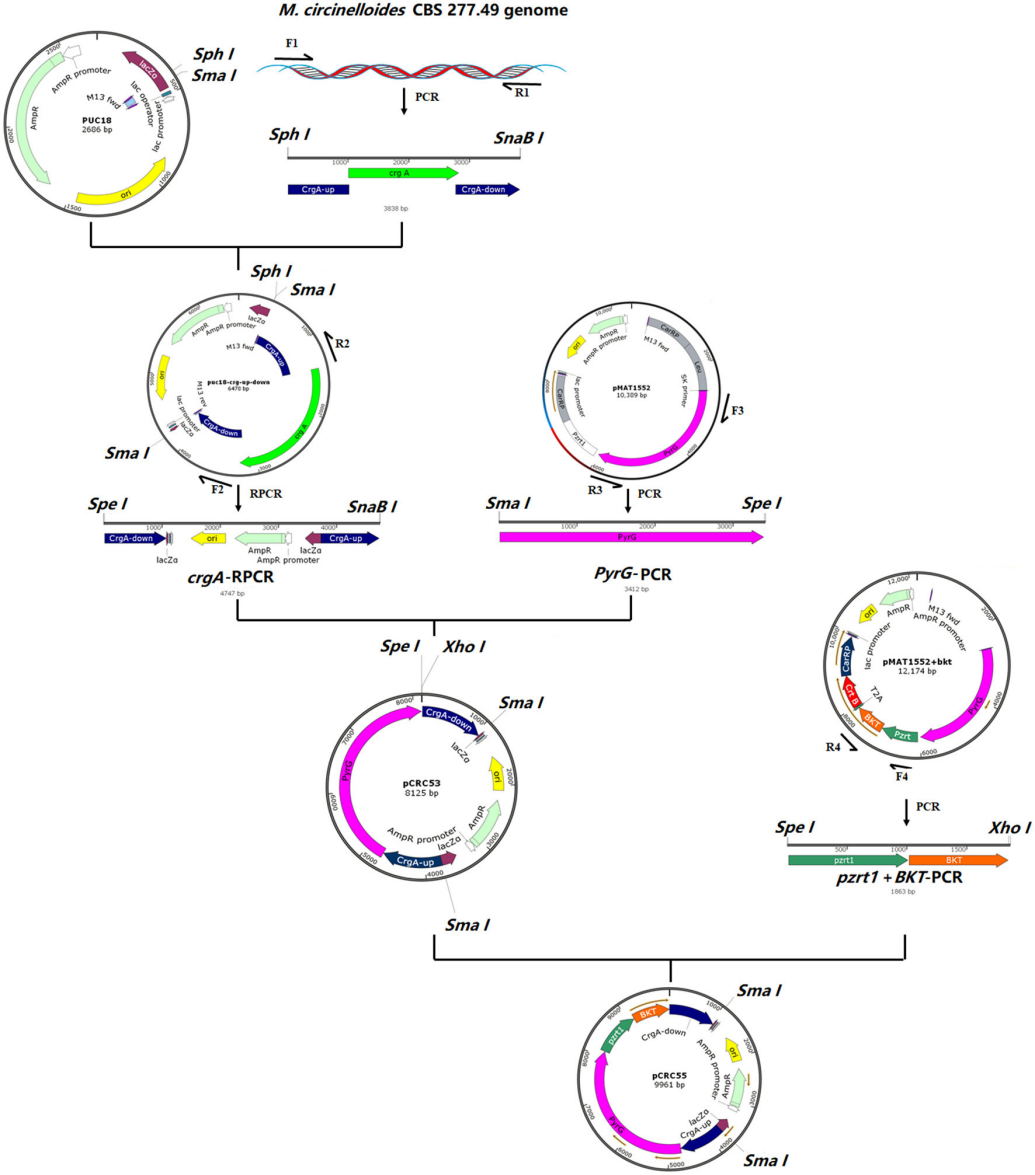
The structure of plasmids pCRC53 and pCRC55. The gene *bkt* was isolated by PCR amplification with specific primers. The PCR fragments were ligated by T4 DNA ligation to generate these plasmids.

For the construction of pCRC53, *crgA* gene (JGI accession number: 39344) with 1 kb up- and down-stream region was amplified from the genome of *M. circinelloides* CBS 277.49 by PCR using the primer pair F1/R1 (Supplementary Table S2). The *crgA* fragment was digested with *SphI* and *SnaBI* and the pUC18 vector was digested with *SmaI* and *SphI* restriction enzymes followed by ligation of these two linear fragments by T4 DNA ligase. From this ligated circular vector, *crgA* coding sequence was deleted by performing reverse PCR (RPCR) using primers F2/R2, resulting in a linear fragment of pUC18 that contained *crgA* up-and down-stream sequences. This linear fragment was then digested with *SpeI* and *SnaBI* restriction endonuclease. The *pyrG* fragment was amplified from the plasmid pMAT1552+bkt using primers F3/R3 and digested with *SmaI* and *SpeI*. Then both the digested fragments were ligated by T4 DNA ligase to make the basic plasmid pCRC53, which was used for the construction of recombinant plasmid pCRC55 carrying the *bkt* gene. For this, the joined *PzrtI* and *bkt* fragment was isolated from modified plasmid pMAT1552+*bkt* using primers F4/R4 as mentioned in Supplementary Table S2. Then pCRC53 was digested with *SpeI* and *XhoI*. The PCR amplified product containing joined *Pzrt1* and *bkt* was also digested with *SpeI* and *Xhol* and then ligated by T4 DNA ligase to obtain the final recombinant plasmid pCRC55. The chemically competent *E. coli* DH5α was used for maintaining and propagating recombinant plasmids during gene cloning experiments. The presence of recombinant plasmids in these *E. coli* cells was confirmed by DNA sequencing (Sangon Biotech, Shanghai Co., Ltd, China). The steps of the gene cloning strategy with the complete map of pCRC53 and pCRC55 are presented in Figure 1.

Both pCRC53 and pCRC55 were digested with *SmaI* and *AatII* to release 5.45 and 7.29 kb fragments, respectively, containing overexpression cassettes, which were used to transform MU402 double auxotrophic strain as described previously (17). In all the transformation experiments, transformants were selected based on auxotrophy complementation and change in color under dark conditions (18).

### Transformation Experiment

For electroporation-mediated transformation, the previously described procedure was followed with some minor modifications (17). Fresh spores of double auxotrophic strain MU402, grown on YPG media for 5–7 days were harvested and nearly 12.5 × 10^7^ spores were inoculated into 25 mL YPG media supplemented with uridine and leucine and placed at 4°C overnight without shaking and then incubated at 28°C at 200 rpm to get germinated spores (2–4 h). Germinated spores were recovered at room temperature by centrifugation at 1,100 rpm for 5 min and then washed with ice-cold phosphate buffer. For protoplast generation, germinated spores were resuspended in 5 ml of PS buffer treated with 0.3 μL chitosanase RD (C0794, Sigma–Aldrich) and 1 mg/mL lysing enzymes (L-1412; Sigma–Aldrich). Removal of the cell wall was assured by using a phase-contrast microscope to monitor the loss of cell wall-associated refringence in the protoplast. Then the prepared protoplasts were suspended in 800 μl of 0.5 M sorbitol, which could be used for electroporation of eight different transformations.

For electroporation, 4–10 μg of linear DNA fragment in a volume of 10–20 μL was added to 100 μL of protoplasts and electric pulse was applied by setting the following condition of electroporator: field strength of 0.8 kV, constant resistance of 400 Ω, and capacitance of 25 μF. After electroporation, 1 ml of ice-cold YPG was added immediately and the solution mixture was incubated at 26°C and 100 rpm for 1 h. Then centrifugation was done to recover protoplasts, followed by resuspension in 500 μl of YNB plus 0.5 M sorbitol and spreading on selective medium (MMC plus 0.5 M sorbitol). These plates were covered with aluminum foil and placed in an incubator at 28°C for 3–4 days.

### Molecular Techniques

Extraction and purification of plasmid DNA were performed with the plasmid mini kit and cycle pure kit (Omega-Biotek, Norcross, United States). PCR products were purified using an Exbio PCR Product purification kit according to the instructions of the manufacturer. For the preparation of genomic DNA, mycelium was disrupted with a pestle and mortar in liquid nitrogen, and DNA was extracted using DNA quick Plant system kit (Tiangen Biotech Co., Ltd., Beijing, China) according to the instructions of the manufacturer. DNA fragments were purified from agarose gel using the Gel Extraction Kit (Omega Biotek, Norcross, United States).

### Determination of CDW, Glucose, and Nitrogen Concentration

Control and overexpressing strains were grown in a 2 L fermenter containing a 1.5 L modified K&R medium. Mycelia of both strains were collected at 3, 6, 9, 12, 24, 48, 72, and 96 h from the fermenters for analysis. Biomass was harvested on a dried and pre-weighed filter paper by filtration through a Buchner funnel under reduced pressure and washed three times with distilled water, frozen overnight at −80°C, and then freeze dried. The weight of the biomass was determined gravimetrically. A glucose oxidase Perid-test kit was used to determine the concentration of glucose in the culture media according to the instructions of the manufacturer (Shanghai Rongsheng Biotech Co., Ltd.). For ammonium concentration, the indophenol method was used (19).

### Carotenoid Extraction and Quantification

Carotenoid was extracted as described in our previous study (20). High-performance liquid chromatography (HPLC) was performed for carotenoid quantification in samples. Samples in a volume of 10 μL were loaded on an infinity Lab Proshell 120 EC-C18 column (4.6 × 150, ODS 4 μm). Two solvents A (96% methanol) and B (100% methyl-terc-butyl ether) were used as mobile phase in the following gradient to analyze carotenoid: min/solvent A%/solvent B% was (0/99/1; 8/60/40; 13/46/54; 15/0/100; 18/0/100; 21/99/1; 25/99/1) at a flow rate of 1 ml/min. Column thermostat temperature was set as 35°C and detection wavelength was set as 450 nm, using a diode-array detector (Agilent Technologies, Santa Clara, CA, United States). The following standards were used to identify the carotenoids in transformants: β-carotene, canthaxanthin, astaxanthin, echinenone (Sigma–Aldrich). The total carotenoids were quantified by a spectrophotometer at 450 nm and measured using an extinction coefficient of 2,500 (A1% = 2,500) as previously described (21).

### RNA Extraction and Analysis of Genes Expression

For RNA extraction, Mc-55 was grown in a 2 L fermenter with 1.5 L K&R media, and biomass were collected at 3, 24, 48, and 72 h. Total RNA was extracted by using TRIzol after disruption of biomass in pestle and mortar, using liquid nitrogen as described previously (22). RNA was reverse transcribed using the Prime ScriptRT reagent kit (Takara Biotechnology, Dalian Co., Ltd, Dalian, China) according to the instructions of the manufacturer. To investigate the expression levels of the canthaxanthin biosynthesis gene *bkt*, real-time quantitative PCR (RT-qPCR) was conducted using specifically designed primers (Supplementary Table S2) on Light Cycler 96 Instrument (Roche Diagnostics GmbH, Switzerland) with FastStart Universal SYBR Green Master (ROX) Supermix (Roche) according to the instructions of the manufacturer. The mRNA expression level was normalized to levels of actin gene and the results were determined as relative expression levels. The data were quantified by the method of 2–ΔΔ^Ct^.

### Statistical Analysis

The mean values were calculated from the data obtained from three independent experiments. Statistical analysis was conducted by one-/two-way ANOVA with multiple comparison tests wheresoever applicable. These calculations were done by the GraphPad Prism software for Windows (San Diego, CA, United States) and *p* < 0.05 was considered as statistically significant.

## Results

### Generation of *bkt* Overexpressing Strains of *M. circinelloides*

To overexpress the *bkt* gene in *M. circinelloides* CBS 277.49, an expression vector pCRC55 was constructed to allow targeted integration of *bkt* in the *crgA* locus. The strong promoter *zrt1* was used for the overexpression of *bkt* gene. Basic plasmid pCRC53 was constructed to disrupt *crgA* locus and act as a control plasmid to generate control strain Mc-53. Both basic pCRC53 and recombinant plasmids pCRC55 were digested with *SmaI* and *AatII* to obtain linear fragments. These linear fragments were then used to transform the recipient strain MU402 generating a control strain named Mc-53 and recombinant strain Mc-55, respectively. Two independent *bkt* overexpressing transformants, named Mc-55, Mc-55-1 were selected.

**Figure 2:**
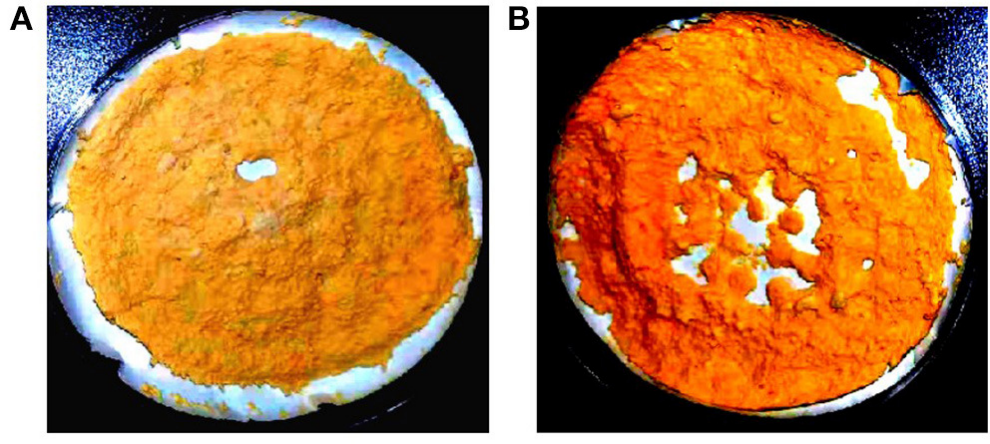
Lyophilized samples of *Mucor circinelloides:* **(A)** control strain Mc-53 and **(B)** *bkt* overexpressing strain Mc-55 grown on K&R media.

Due to the syncytial nature of *Mucor* hyphae, and the multinucleate nature of protoplasts (23), and spores, heterokaryotic transformants were obtained initially. Therefore, transformants were grown on selective media for several consecutive vegetative cycles to obtain stable *pyrG*+ transformants. After more than 10 vegetative cycles, homokaryotic transformants were obtained for both the linear fragments of plasmids pCRC53 and pCRC55. The successful expression of *bkt* gene in *M. circinelloides* was confirmed by the accumulation of ketocarotenoids such as echinenone, canthaxanthin, and astaxanthin in small amounts. Gene replacement in the *crgA* locus resulted in deep yellow colonies that are recognizable among the pale-yellow colonies of wild-type *M. circinelloides* in dark. This color change was an indication of a higher amount of β-carotene accumulation due to disruption of *crgA* gene locus, resulting in deep yellow mycelia for control strain Mc-53 and reddish-orange in case of overexpressing strain Mc-55 in dark (Figure 2). Lack of *crgA* function is associated with enhanced production of carotene under both light and dark conditions (24).

**Figure 3:**
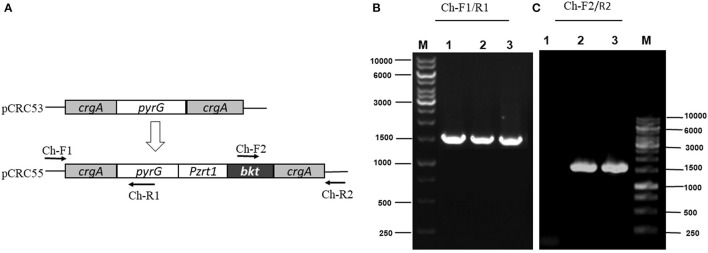
PCR amplification of genomic DNA of control (Mc-53) and recombinant strains (Mc-55) with the indicated primers. Lane 1 representing the control strain and Lane 2, 3 showing the presence of *bkt* in recombinant strains Mc-55 and Mc-55-1, respectively. **(A)** Plasmid structure of pCRC53 and pCRC55 for *bkt* overexpression. **(B)** Verification of the presence of marker *pyrG* inside transformants. Lane M: Marker, 1: control strain, 2-3: Recombinant strains (Ch-F1/R1primers). **(C)** Verification of the presence of *bkt* inside transformants. Lane M: Marker, 1: control strain, 2-3: Recombinant strains (Ch-F2/R2 primers).

The integration of the exogenous gene in all transformants was confirmed by PCR amplification using primers (Ch-F1/R1 and Ch-F2/R2) listed in Supplementary Table S2. Genomic DNA was used as a template. The first primer pair (Ch-F1/R1) was designed in such a way that it could amplify few base pairs (bp) beyond *crgA* 1 kb upstream (inside genome) to some portion of *pyrG* (Figure 3A). This primer pair amplified the expected size, 1,513 bp sequence from up-streams of *crgA* verifying the presence of marker *PyrG* inside the genome. The same band size was also obtained for Mc-53 as our control strain also contained *pyrG* selection marker (Figure 3B). The other primer pair (Ch-F2/R2) was designed to amplify a region of 54 bp beyond *crgA* 1 kb downstream to almost half of the segment of the exogenous *bkt* gene. Amplification reaction produced an expected 1,533 bp fragment as shown in Figure 3C, which confirmed the presence of *bkt* gene inside the overexpressing strain Mc-55 while no fragment was amplified for control strain Mc-53 (Figure 3C). Thus, PCR amplification results confirmed the integration of the target gene into the genome of the transformants. The presence of a single band indicated the homokaryotic nature of the transformants.

Two overexpression strains Mc-55, Mc-55-1, and one control strain Mc-53 were grown in 150 mL K&R medium for 3–4 days in 500 ml baffled flasks to perform additional screening. Since CDW and canthaxanthin of these two strains were comparable (data not shown), only one strain Mc-55 was selected for further experiments.

### Growth of *bkt* Overexpressing Strain

**Figure 4:**
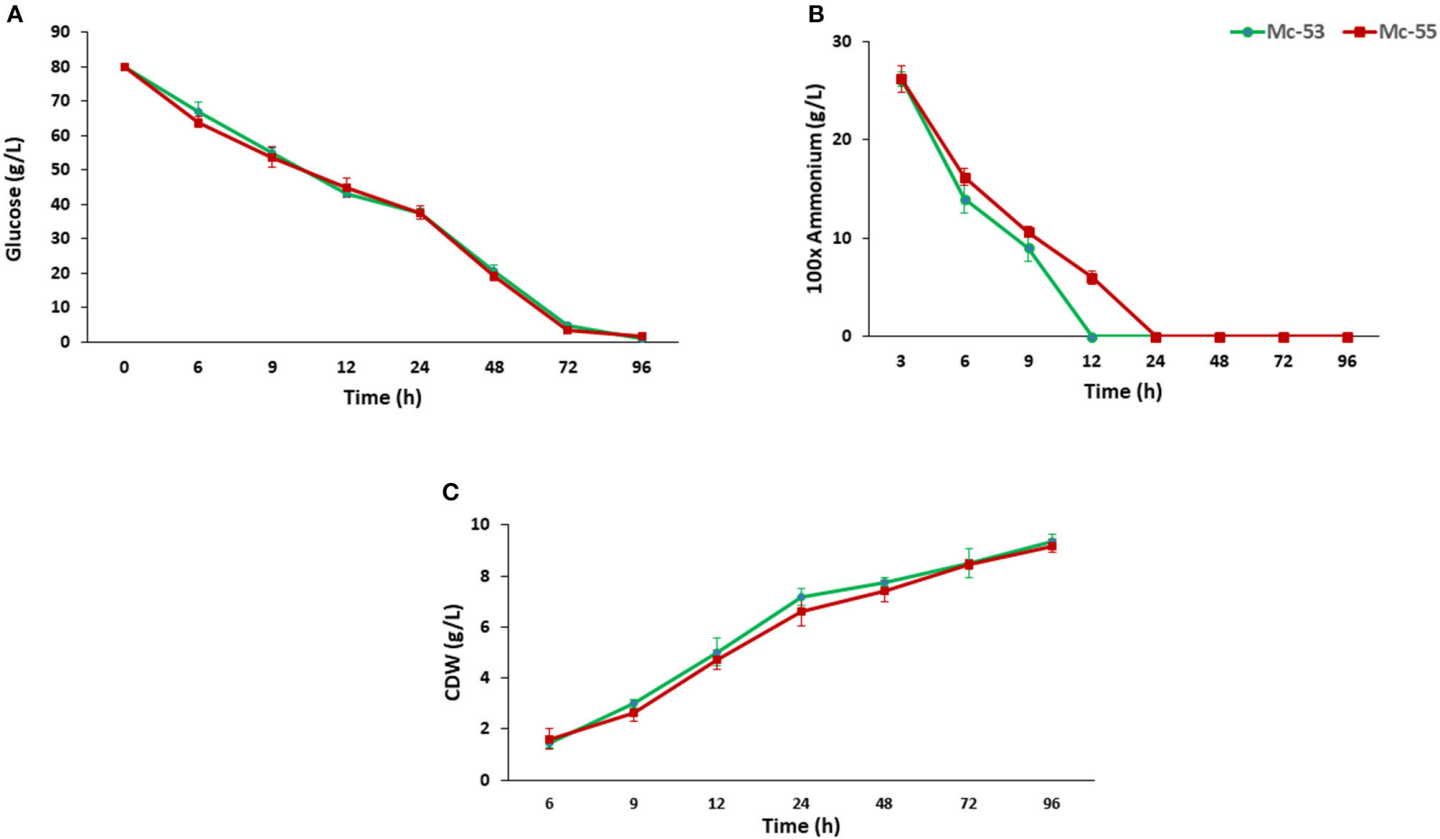
Determination of glucose and nitrogen consumption and cell CDW in recombinant strain Mc-55 and control strain Mc-53 at indicated time intervals. **(A)** Glucose concentration, **(B)** ammonium concentration, and **(C)** CDW. Values were the mean of three independent experiments. Error bars represent the standard deviation of the data.

CDW and consumption of ammonium and glucose by Mc-55 were analyzed and compared with control strain Mc-53 as shown in Figure 4. In general, both the control and the recombinant strain showed a similar and typical growth profile. Ammonium was completely exhausted in 12–24 h in Mc-55 but it was consumed more rapidly by the control strain Mc-53, while glucose remained in sufficient amount during the entire fermentation time (Figures 4A,B). CDW was increased rapidly until 24 h and slowed down (Figure 4C). However, cell growth was slightly affected in both strains as compared to wild-type CBS 277.49, which might be attributed to *crgA* disruption in these strains.

### Carotenoid Accumulation in *bkt* Overexpressing Strain

Detailed carotenoid profile of recombinant strain is presented in Table 1. The mycelia were collected from fermenters at the specified intervals and disrupted using pestle and mortar into fine powder for carotenoid extraction. HPLC analysis showed β-carotene, canthaxanthin, echinenone as major carotenoids in Mc-55. While in the control strain only β-carotene was observed as a major carotenoid, verifying that *bkt* gene was successfully expressed in Mc-55. Nevertheless, the control strain Mc-53 produced an elevated level of β-carotene (2211 ± 47 μg/g of CDW), which was 8-folds higher as compared to recipient strain MU402, that could only produce 275 ± 13 μg/g of β-carotene under the same cultivation condition (data not shown). This substantial increase in β-carotene production could be possible due to *crgA* disruption in Mc-53 since disruption of *crgA* caused over-accumulation of β-carotene even in dark by increasing the mRNA levels of *carB* and *carRP* (24).

**Table 1:** Total carotenoid content and major carotenoid composition of control Mc-53 and recombinant Mc-55 strains.

**Time (h)**	**β-Carotene**	**Echinenone**	**Canthaxanthin**	**Astaxanthin**	**Total carotenoids**
**Mc-53**					
6	169 ± 13	**–**	**–**	**–**	254 ± 44
9	290 ± 28	**–**	**–**	**–**	388 ± 28
12	523 ± 16	**–**	**–**	**–**	701 ± 16
24	952 ± 25	**–**	**–**	**–**	1197 ± 73
48	1,673 ± 40	**–**	**–**	**–**	1877 ± 45
72	2211 ± 47	**–**	**–**	**–**	2495 ± 56
96	2167 ± 16	**–**	**–**	**–**	2424 ± 37
**Mc-55**					
6	33 ± 14	61 ± 9	63 ± 13	2 ± 0.9	291 ± 48
9	54 ± 22	87 ± 15	105 ± 22	5 ± 1	440 ± 36
12	118 ± 12	143 ± 8	178 ± 29	8 ± 1.3	611 ± 32
24	207 ± 22	244 ± 30	381 ± 23	14 ± 1.4	985 ± 26
48	324 ± 21	359 ± 25	507 ± 12	27 ± 1.8	1470 ± 54
72	422 ± 23	410 ± 29	576 ± 28	41 ± 2.3	1789 ± 47
96	391 ± 14	403 ± 25	556 ± 13	38 ± 2.2	1743 ± 31

*The indicated amounts are average values in μg/g of cell dry weight from the data of three independent experiments. Carotenoid extraction was carried out after cultivation of the strains in K&R media for 4 days at 28°C under continuous light*.

**Figure 5:**
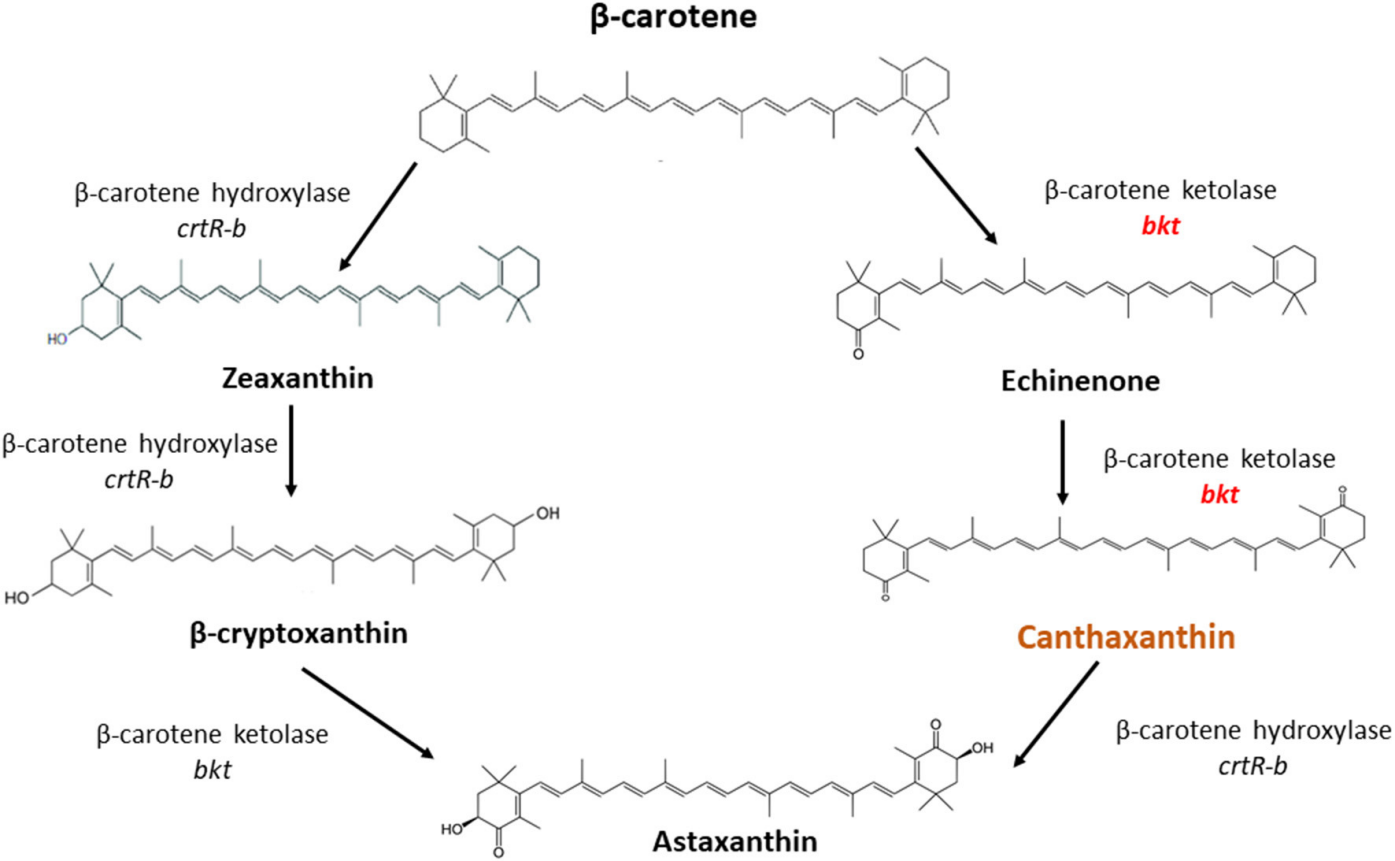
The possible pathway for conversion of β-carotene to canthaxanthin and astaxanthin in *M. circinelloides* as proposed by Misawa et al. (25).

Overexpression of the algal *bkt* in the Mc-55 gene led to the production of ketoderivatives (Figure 5). The accumulation of canthaxanthin started at 6 h and substantially increased with the progress of cultivation time. Mc-55 produced the maximum amount of canthaxanthin at 72 h. Other measured carotenoids and total carotenoids also showed the same trend. The highest amount of canthaxanthin (576 ± 28 μg/g) and echinenone (410 ± 29 μg/g) and a comparatively small amount of astaxanthin (41 ± 2.3 μg/g) were produced, which corresponded to 32, 23, and 3% of total carotenoid in Mc-55 at 72 h, respectively. This significant amount of canthaxanthin production might be possible in the current study due to the availability of a higher amount of β-carotene substrate as it is the closest intermediate to canthaxanthin in the introduced pathway (Figure 5).

### The Expression Level of *bkt* Gene in Overexpressing Strain

**Figure 6:**
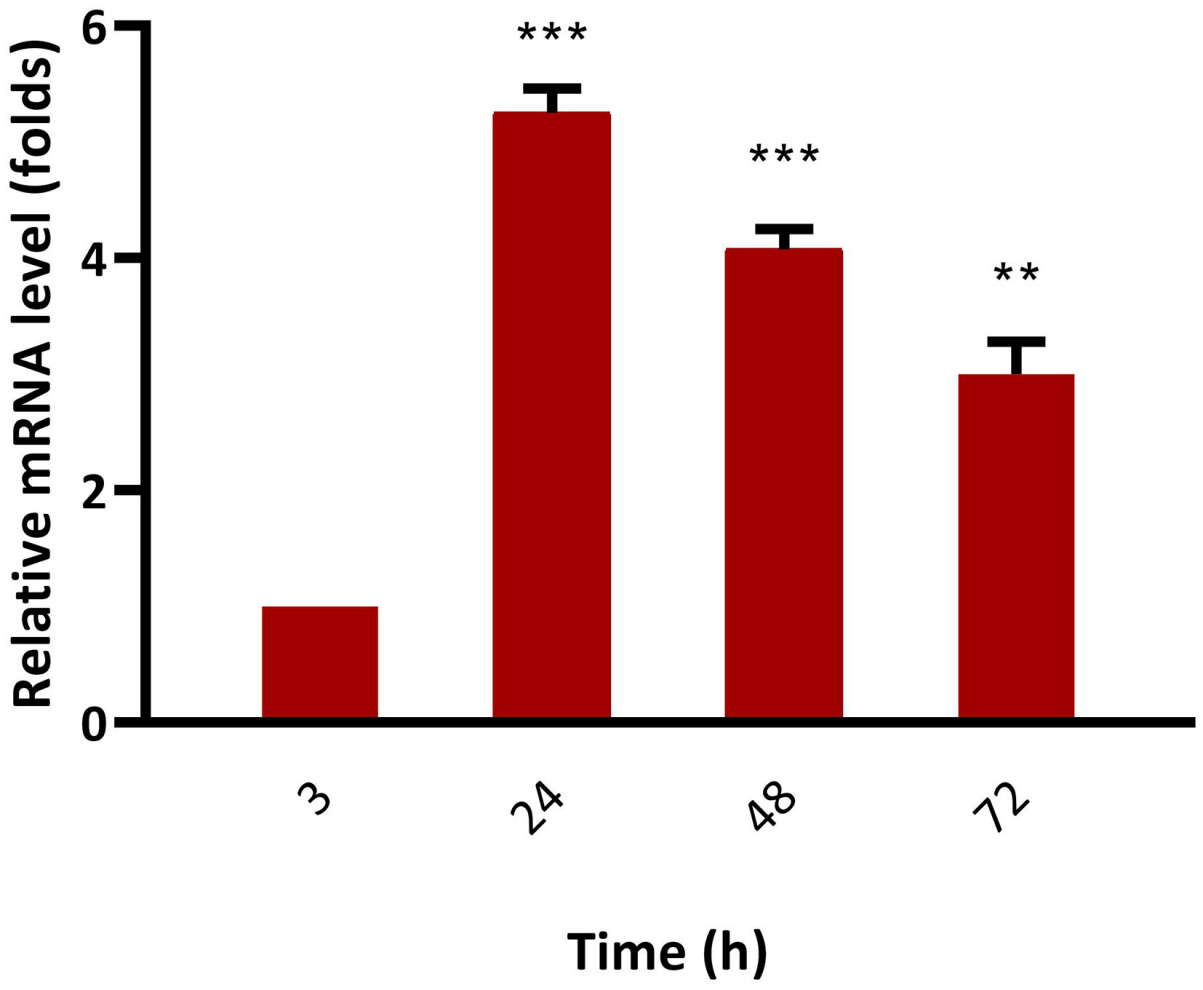
Determination of expression levels of *bkt* genes by RT-qPCR in the overexpressing strains Mc-55. Values were the mean of three independent experiments. Error bars represent the standard deviation of the data. Asterisks indicate that the difference is significant.***p < 0.001 in comparison between 3, 24, and 48 h, and **p < 0.01 in comparison between 24, 48, and 72 h of growth.

The mRNA expression level of *bkt* in overexpressing strain Mc-55 was analyzed by RT-qPCR at specified intervals. In control strain Mc-53, the mRNA level of *bkt* was undetectable throughout the culture time. The mRNA expression level of Mc-55 was considered as 1 at 3 h, and by comparing with this value the expression level was determined at other time intervals. The expression level of Mc-55 was quickly increased at the start of fermentation till 24 h but showed a decreasing trend afterward. It was observed that the expression level of *bkt* was increased by 5.3-, 4.1-, and 3-folds at 24, 48, and 72 h, respectively, and maintained at elevated levels throughout the cultivation time, suggesting that it was overexpressed successfully in Mc-55 (Figure 6).

## Discussion

In *M. circinelloides* β-carotene is produced as a major carotenoid *via* the mevalonate pathway along with a lower amount of zeaxanthin and β-cryptoxanthin due to weak hydroxylase activity. However, no astaxanthin could be produced due to the absence of ketolase enzymes (11). β-carotene can be further converted into canthaxanthin by β-carotene ketolase encoded by gene *bkt* in algae and *crtW* in bacteria, which add keto group in the ring of β-carotene at 4,4-position. The efficient conversion of β-carotene into canthaxanthin depends on the ketolase enzyme with higher catalytic activity. *Haematococcus pluvialis* is a marine, astaxanthin producing algae, in which the conversion of β-carotene to astaxanthin is carried out by the enzymes β-carotene ketolase and hydroxylase with canthaxanthin as an intermediate compound (Figure 5). *Haematococcus pluvialis*, being a natural source of valuable xanthophylls, is grown for ketocarotenoid production, but commercial-scale cultivation of this algae is embedded with few drawbacks. For example, high light requirement, slow growth at room temperature, as well as vulnerable to contamination by other algae (26). Over the past two decades, *M. circinelloides* has been successfully engineered for various useful metabolites by either overexpressing indigenous genes or by introducing the exogenous biosynthetic genes (11, 27–30).

In the present study, *bkt* gene from *H. pluvialis* was successfully overexpressed in *M. circinelloides* under the control of strong promoter *zrt1* to construct the canthaxanthin biosynthesis pathway. The successful integration of *bkt* was indicated by the accumulation of ketocarotenoid in Mc-55, producing ~576 ± 28 μg/g canthaxanthin, which is the highest titer of canthaxanthin ever reported in *M. circinelloides*. This amount was almost 3-folds more than the canthaxanthin content of the wild-type strain of *Gordonia jacobaea* (200 μg/g) (31), but almost comparable with 600 μg/g of canthaxanthin *in Brevibacterium* KY-4313 (32), both of them are considered as a natural source of canthaxanthin. Similarly, the bacterium *Dietza natronolimnaea* HS-1 was reported to produce a maximum of 8,923 ± 18 μg/L canthaxanthin in a fed-batch process (33). Previously, Papp et al. had successfully engineered *crtW* gene in *M. circinelloides* from marine bacterium but the amount of canthaxanthin produced was very low, i.e., 6–13 μg/g (12). The transformants obtained in our study accumulated a significantly higher amount of canthaxanthin than that obtained by a previous study in which a maximum of 443 ± 71 μg/g canthaxanthin was produced by *Mucor* after co-expression of *crtW and crtZ* and cultivation of the mutant strain on the combination of glucose and dihydroxyacetone (34). Similarly, in another report overexpression of *crtW* gene from *Paracoccus* into *M. circinelloides* produced a maximum amount of canthaxanthin in the range of 100–240 μg/g after genetic modification and medium optimization (13). Csernetics et al. also introduced two genes *crtR* and *crtS* from the yeast *Xanthophyllomyces dendrorhous* in *M. circinelloides*, which are responsible for the conversion of β-carotene to astaxanthin. After laborious genetic modification, a maximum of 190 μg/g of canthaxanthin was produced in a mutant strain of *M. circinelloides* (29). So, the higher production of canthaxanthin could be attributed to the higher catalytic efficiency of *bkt* from an algal source in comparison to its bacterial and yeast counterpart.

However, control strain Mc-53 in the present study showed superior production of β-carotene up to 2211 ± 47 μg/g in the fermenter at 72 h with increased total carotenoid as compared to *bkt* overexpressing strain Mc-55 (Table 1). In overexpressing strain Mc-55, the concentration of β-carotene was drastically reduced to 422 ± 23 at 72 h which might be attributed to the conversion of β-carotene into ketocarotenoids such as echinenone and canthaxanthin. This could also be explained as a possible feedback effect of β-carotene and its derivatives produced during the β-carotene biosynthesis. So, it might be possible that β-carotene production has decreased in Mc-55 due to its conversion into canthaxanthin and affected the functioning of genes involved in carotenogenesis (13).

The successful overexpression of the *bkt* gene from an algal source was also verified by RT-qPCR. The result showed that mRNA levels of the *bkt* gene remained elevated throughout the fermentation time. The expression level of *bkt* showed maximum expression reaching up to 5.3-folds at 24 h after which a decrease in the expression level was observed. However, the maximum production of canthaxanthin was achieved in our study at 72 h, which might be explained as *bkt* gene regulated the canthaxanthin accumulation by post-transcriptional or post-translational regulatory processes (35).

In our study, the higher canthaxanthin production might be possible because of two reasons (1): The expression of codon-optimized *bkt* gene under the control of strong promoter *zrt1*; (2): Availability of a high amount of β-carotene precursor, achieved by the disruption of *crgA*, a well-known repressor of carotenogenesis (24). Codon optimization of the algal *bkt* gene was done with the codon preference of *M. circinelloides*. Zhou et al. also used codon-optimized genes for astaxanthin synthesis by *Saccharomyces cerevisiae* and found increased expression levels of *bkt* and *crtZ* (36) as compared to the non-optimized aforementioned gene sequence. Moreover, it is well-known from the literature that initial astaxanthin synthesis utilizes the existing β-carotene as a precursor, so the production of the bulk astaxanthin esters depends on *de novo* β-carotene synthesis (37). *CrgA* is identified as a repressor of carotenoid biosynthesis in *M. circinelloides* because disruption of *crgA* caused over-accumulation of β-carotene even in dark by increasing the mRNA levels of structural carotenogenic genes, that is, *carB* and *carRP* (24). The *crgA* gene suppresses carotenoid biosynthesis in *Mucor* by proteolysis-independent mono-ubiquitilation and di-ubiquitilation of white collar *wc-1b*, which triggers the transcription of *carB* and *carRP* genes when non-ubiquitilated (38). A previous study has also shown that the deletion of *crgA* resulted in higher lycopene accumulation in mutant *M. circinelloides* strain (39). Similarly, Zhang et al. found that disruption of *crgA* caused over-accumulation of β-carotene in mutant strains of *M. circinelloides* (18).

The canthaxanthin-producing strain of *M. circinelloides* obtained in our study can be a strong putative candidate for industrial production because of two main reasons; first, the fermentation process using *M. circinelloides* would be much simpler than the complicated growth requirement of *H. pluvialis*. Second, dimorphic nature favors its biotechnological application. The highest canthaxanthin production of 576 ± 28 μg/g was achieved in *Mucor*, which is the maximum reported canthaxanthin in *Mucor* to date. This study can be regarded as a good extension of previous genetic modifications done in *M. circinelloides* for canthaxanthin production. In conclusion, canthaxanthin production in our recombinant strain could be further augmented by improving cultivation conditions or by employing a fed-batch process to enhance the biomass productivity for the volumetric production of canthaxanthin. Genetic engineering of early steps of the mevalonate pathway to increase the supply of rate-limiting precursor, suppressing the competitive fatty acid and sterol biosynthesis pathway, and improving the fermentation process could be attractive strategies for future improvement of canthaxanthin production in the obtained recombinant strains of *M. circinelloides*.

## Data Availability Statement

All data generated or analyzed during this study are included in this published article and its Supplementary Material.

## Author Contributions

TN and JY performed the experiments and manuscript writing. SN worked in experimental design. CS reviewed the initial draft and helped in editing. YN and HM were involved in the experimental design and data analysis. AF and SL helped with the HPLC analysis and quantification. SU helped in statistical analysis. WY helped in drawing images. VG involved in results interpretations. YS proposed the project and was involved in data analysis, and review of the final draft. All authors read and approved the final manuscript.

## Funding

This work was supported by the National Natural Science Foundation of China (Grant Nos. 31670064 and 31972851), the TaiShan Industrial Experts Program tscy 20160101, and the Shandong provincial key technology R&D plan (2018GNC110039, 2018GSF121013).

## Conflict of Interest

The authors declare that the research was conducted in the absence of any commercial or financial relationships that could be construed as a potential conflict of interest.

## Publisher's Note

All claims expressed in this article are solely those of the authors and do not necessarily represent those of their affiliated organizations, or those of the publisher, the editors and the reviewers. Any product that may be evaluated in this article, or claim that may be made by its manufacturer, is not guaranteed or endorsed by the publisher.
