# Supplementary material for: Genetic Modification of Mucor circinelloides for Canthaxanthin Production by Heterologous Expression of β-carotene Ketolase Gene
Source: Front Nutr. 2021 Oct 13;8:756218. doi: 10.3389/fnut.2021.756218 (PMC8548569; doi:10.3389/fnut.2021.756218)
Supplement: Supplementary file 1 [file Data_Sheet_1.docx]

Optimized DNAof Bkt

GenBank accession #: MZ020513

ATGCCTTCTGAATCTTCTGATGCTGCTAGACCTGTCTTGAAGCATGCTTACAAGCCTCCTGCTTCTGATGCTAAGGGTATTACTATGGCTTTGACCATTATTGGTACTTGGACTGCTGTCTTTTTGCATGCTATCTTTCAAATTCGTTTGCCTACTTCTATGGATCAATTGCATTGGTTGCCTGTCTCTGAAGCTACTGCTCAATTGTTGGGTGGTTCTTCTTCTTTGTTGCATATCGCTGCTGTCTTCATTGTCTTGGAATTCTTGTACACCGGCTTGTTCATTACTACCCATGATGCTATGCATGGTACTATTGCTTTGAGAAACAGACAATTGAACGATTTGTTGGGTAACATTTGTATTTCTTTGTACGCTTGGTTTGATTACTCTATGCATTGGGAACATCATAACCATACTGGTGAAGTCGGTAAAGATCCTGATTTCCATAAGGGTAATCCTGGTTTGGTCCCTTGGTTCGCTTCTTTCATGTCTTCTTACATGTCTTTGTGGCAATTTGCTCGTTTGGCTTGGTGGGCTGTCGTCATGCAAACTTTGGGTGCTCCTATGGCTAACTTGTTGGTCTTCATGGCTGCTGCTCCTATTTTGTCTGCTTTTCGTTTGTTTTACTTCGGTACCTACTTGCCTCATAAGCCTGAACCTGGTCCTGCTGCTGGTTCTCAAGTCATGTCTTGGTTCAGAGCTAAGACCTCTGAAGCTTCTGATGTCATGTCTTTTCTGACTTGTTACCATTTCGATTTGTTCGCTCCTTGGTGGCAATTGCCTCATTGTCGTCGTTTGTCTGGTCGTGGTTTGGTCCCCGCTTTGGCT
