# Supplementary material for: Genetic Modification of Mucor circinelloides for Canthaxanthin Production by Heterologous Expression of β-carotene Ketolase Gene
Source: Front Nutr. 2021 Oct 13;8:756218. doi: 10.3389/fnut.2021.756218 (PMC8548569; doi:10.3389/fnut.2021.756218)
Supplement: Supplementary file 2 [file Table_1.docx]

**Supplementary files:**

**Table S1**: List of plasmids used in this study.

| **Plasmid** | **Description** | **Source/ reference** |
| --- | --- | --- |
| pUC18 | General cloning vector (Amp) | Fermentas |
| pEUKA4-gox1 | Source of strong promoter *Pgpd1* (Amp) | AJ305345.1 |
| pMAT1552 | pyrG gene of *M. circinelloides* (Amp), strong promoter Pzrt1 | (Khan *et al.*, 2019) |
| pCRC53 | 1 kb up-down of *crgA* gene with pyrG (Amp) | This study |
| pCRC55 | *bkt* , *Pzrt1* with flanking regions of *crgA* (Amp) and pyrG | This study |

**Table S2.** Primers used in this study.

| **Primers** | **Sequence (5′-3′)** | **Applications** |
| --- | --- | --- |
| F1 | ATGCTGCATGCCCCGGGATTCCACACATTAGTAAACGAG | Gene cloning |
| R1 | GCTATTACGTACCCGGGGATCATTCAGCAGATTAGACG | Gene cloning |
| F2 | TGATCACTAGTTATACTCGAGCGTCCAATCCAGCTCAACC | Gene cloning |
| R2 | TACGTTACGTAGCACTTTTACGAAATGTGCTTTC | Gene cloning |
| F3 | TGCATCCCGGGACCGGGCAGAATGATTAACAAGG | Gene cloning |
| R3 | TGATCACTAGTTAAAAGGATCCAGAGGCGCTAAAAAGTG | Gene cloning |
| F4 | CTGCAACTAGTATCATCATCGATGTTTGTGCTG | Gene cloning |
| R4 | AGTATCTCGAGTCAAGCCAAAGCGGGGACCAAAC | Gene cloning |
| RT-bkt-F | AGCCTCCTGCTTCTGATGCTAAGG | RT-qPCR |
| RT-bkr-R | GAAGAAGAACCACCCAACAATTGAGC | RT-qPCR |
| Act-F | GATGAAGCCCAATCCAAGAGAGGT | RT-qPCR |
| Act-R | TCTTCTCACGGTTGGACTTGGG | RT-qPCR |
| chk-F1 | GCTATTCAAGGTCAGGGCGTC | Gene confirmation |
| chk-R1 | GAAGAAGATGTGTTGTGGTGGCG | Gene confirmation |
| chk- F2 | GTAACATTTGTATTTCTTTGTACGC | Gene confirmation |
| chk-R2 | AGACATCAAAAGCTGCATACGGG | Gene confirmation |
